# Local and regional anaesthesia in dogs and cats: Descriptions of specific local and regional techniques (Part 2)

**DOI:** 10.1002/vms3.218

**Published:** 2020-01-21

**Authors:** Tamara Grubb, Heidi Lobprise

**Affiliations:** ^1^ Washington State University Pullman WA USA; ^2^ Main Street Veterinary Hospital Flower Mound TX USA

**Keywords:** analgesia, bupivacaine, liposome‐encapsulated, local anaesthetics, local block

## Abstract

Pain management in veterinary patients is a crucial component of appropriate patient care. Local anaesthetic drugs used in local and regional blockade can completely block the transmission of nociceptive impulses, decreasing both intra‐operative nociception and postoperative pain, while decreasing the potential incidence of adverse effects that can be associated with systemic boluses of drugs. For efficacy and safety, this class of drugs is recommended as part of the analgesic protocol in the majority of surgical procedures and traumatic injuries. Numerous local and regional blocks are proven effective in dogs and cats, thus providing the clinician with ample opportunity to include these blocks in practice. This manuscript, Part 2 of a two‐part instalment, focuses on brief descriptions of select commonly used local/regional anaesthesia techniques for dogs and cats that cover a multitude of painful surgeries/injuries and that can be implemented in any practice. In Part 1 of this topic, detailed information on local anaesthetic drugs commonly used in small animal practice was reviewed (Grubb & Lobprise, 2020).

## Graphical abstract

This manuscript, Part 2 of a two‐part instalment, focuses on brief descriptions of select commonly used local/regional anesthesia techniques for dogs and cats that cover a multitude of painful surgeries/injuries and that can be implemented in any practice.

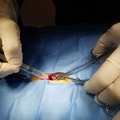

## 1 INTRODUCTION

Due to their ability to profoundly decrease both intraoperative nociception and postoperative pain, local anaesthetic drugs are recommended for use in the majority of surgical procedures and traumatic injuries, as outlined in recent veterinary pain management guidelines (Epstein, 2015; Mathews et al. 2014). Note that ‘pain’ is referred to as ‘nociception’ in anaesthetized patients since a cognitive response, which is prevented by the anaesthetic, is necessary to define pain. Pain is used to describe the sensation in conscious patients. Although perhaps not as easy as an IM or IV injection, in the authors’ opinion and the opinion of other experts (Epstein, 2015) , most local/regional blocks are readily learned and implemented in all types of veterinary practice. This review is written primarily for general practitioners desiring to develop or expand use of local/regional anaesthesia without having to buy specialized equipment, which may require advanced training to maximize use. All local/regional blocks described in this manuscript can be effectively completed using knowledge of dog and cat anatomy, good palpation skills and appropriate injection technique (i.e. gentle insertion of the needle, aspiration prior to injection). This manuscript is not an exhaustive review of all possible blocks or techniques. More advanced techniques (i.e. ultrasound guidance and nerve locators) are mentioned where appropriate but a full description is beyond the desired scope of this paper. These techniques are not necessary for blocks like incision/wound infiltration, peritoneal lavage with local anaesthetics, testicular blocks, etc. However, based on cadaver studies of injectate proximity to nerves (Portela, Verdier, & Otero, 2018 and Portela, Verdier, & Otero, 2012), the accuracy for blocks like brachial plexus, radius/ulnar/median/musculocutaneous (RUMM) and sciatic/femoral may be improved using these techniques. Recommended resources for other block descriptions, more in‐depth information, images and advanced techniques include Campoy & Read, 2013; Otero & Portela, 2018; Portela et al., 2018a and Portela et al., 2018b; and Lerche, Aarnes, Covey‐Crump, & Martinez, 2016.

### 1.1 Drugs, equipment and dog/cat preparation

The drug dosages used by the authors are those listed in the ‘commonly used drugs’ section of Part 1 of this manuscript (Grubb & Lobprise, 2020). However, the volume of drug that can be injected into the tissue is often lower than the mg/kg dose. A suggested drug volume is included for many blocks listed below and is based on drug concentrations of 2% lidocaine, 0.5% bupivacaine, 0.5% ropivacaine and 1.33% bupivacaine liposome injectable suspension (BLIS). However, with all blocks, the total recommended dose of the selected local anaesthetic should be calculated for the species of patient prior to performing each block, and the total amount of local anaesthetic administered should not exceed the calculated dose unless the added dose is insignificant, like the small dose of lidocaine typically used on the arytenoids of cats for intubation. In contrast, as mentioned by the reviewer of this manuscript, the dose of a specific product (not used by the authors) is up to 5 mg/kg for arytenoid desensitization. This is a significant contribution and should be considered as part of the total dose. The drug dosages are listed in Part 1 of this manuscript (Grubb & Lobprise, 2020). The volume of drug (if different from the total dose) and equipment needed to do the blocks are listed in Box 1 and Box 2.

BOX 1 **Equipment and volume of infiltration for dental/oral blocks**

▪ Equipment: Syringes of appropriate size to hold the calculated volume of drug with needles of 25‐, 27‐, or 30‐gauge (non‐deflecting to minimize bending) and of 1‐in to 1 3/8‐in length (Lantz 2003). BLIS is injected through needles 25‐gauge or larger to prevent disruption of the liposomes.
▪ Volume of infiltration (Lantz 2003) :
  - Cats 0.2–0.25 ml per site
  - Dogs 0.25, 0.5, or 1 ml per site for small, medium and large dogs, respectively

BOX 2 **Equipment and volume of infiltration for all other blocks described in this manuscript**

▪ Equipment: Syringes of appropriate size to hold calculated volume of drug and 25‐ to 22‐gauge needles of 1 to 1.5 inches in length. Occasionally, longer needles (2–3 inch) or specialized needles (like epidural needles, e.g. Touhy needles) are required.
▪ Volumes of infiltration: If different from the total calculated volume of drug, volumes are listed with each block.

Unless otherwise stated with a specific block, all of the local anaesthetic drugs discussed in Part 1 (Grubb & Lobprise, 2020) are appropriate for all of the blocks described in this manuscript, but bupivacaine supplied as a liposome‐encapsulated injectable suspension (abbreviated as BLIS in this manuscript; NOCITA®) has some logistical considerations. BLIS is Food and Drug Administration (FDA) approved in the US (and hopefully coming to other countries soon) for injecting into the incisional tissue layers following cranial cruciate ligament surgery in dogs. BLIS is commonly used off‐label in both dogs and cats for tissue infiltration in other surgeries, including amputations and mass removals, and is used for tissue infiltration for wound repair. For these blocks, BLIS is injected just prior to closure of the incision or wound, but not pre‐emptively, as the liposomes could be disrupted by the incision or tissue debridement. Regular bupivacaine can be injected pre‐emptively and BLIS at closure. BLIS is FDA‐approved for peripheral nerve blocks for digit surgery in cats and is commonly used off‐label for numerous other peripheral nerve blocks (e.g. dental/oral, auriculopalpebral, great auricular, brachial plexus [depending on surgical site], RUMM, sciatic, etc.) in both cats and dogs. BLIS can be injected pre‐emptively for these blocks because the liposomes are injected remote to the surgery site and not in a location that would be disrupted by the surgical incision or tissue debridement. Due to their relatively large size, the liposomes release bupivacaine locally rather than diffusing throughout the tissue, thus, BLIS is not likely to be effective for blocks that require migration of the local anaesthetic to fairly distant tissues (e.g. testicular block where the drug injected into the testicle must migrate to the spermatic cord) or where the drug is ‘lavaged’ or ‘splashed’ into a tissue rather than injected into the site where the liposomes can release bupivacaine locally (e.g. peritoneal lavage). See more on BLIS injection technique in Part 1 of this manuscript (Grubb & Lobprise, 2020) and at the product website (NOCITA® injection technique website 2018). Due to the fact that BLIS is fairly new to veterinary medicine and that an encompassing statement regarding BLIS suitability for all blocks described here cannot be made, use of the drug will be specifically addressed in the blocks where appropriate. BLIS is commonly used by the author for all dental/oral blocks so specific statements are not made in that section of blocks.

*Patient preparation:* Wherever possible, the injection site should be clipped and aseptically prepared.

*Precaution:* For all blocks, ASPIRATE prior to injection (potential caveat discussed in the lumbosacral epidural section).

### 1.2 Select local and regional anaesthesia techniques in dogs and cats

All blocks described in this manuscript have been used by the authors unless otherwise indicated. The techniques for some of the blocks are described in other sources, often with detailed diagrams (Duke‐Novakovski, 2016; Lemke & Creighton, 2008; Rioja Garcia 2015; Campoy & Read, 2013; Otero & Portela, 2018). Specific references for block technique and/or efficacy are provided where possible.

### 1.3 General tissue infiltration

*‘Field block’, also called incisional block or ‘line’ block* (Vicente & Bergstrom, 2018; Lascelles, Rausch‐Derra, Wofford, & Huebner, 2016; Yilmaz et al., 2014; Savvas et al. 2008) Figure 1 and Box 3 **.**

**Figure 1:**
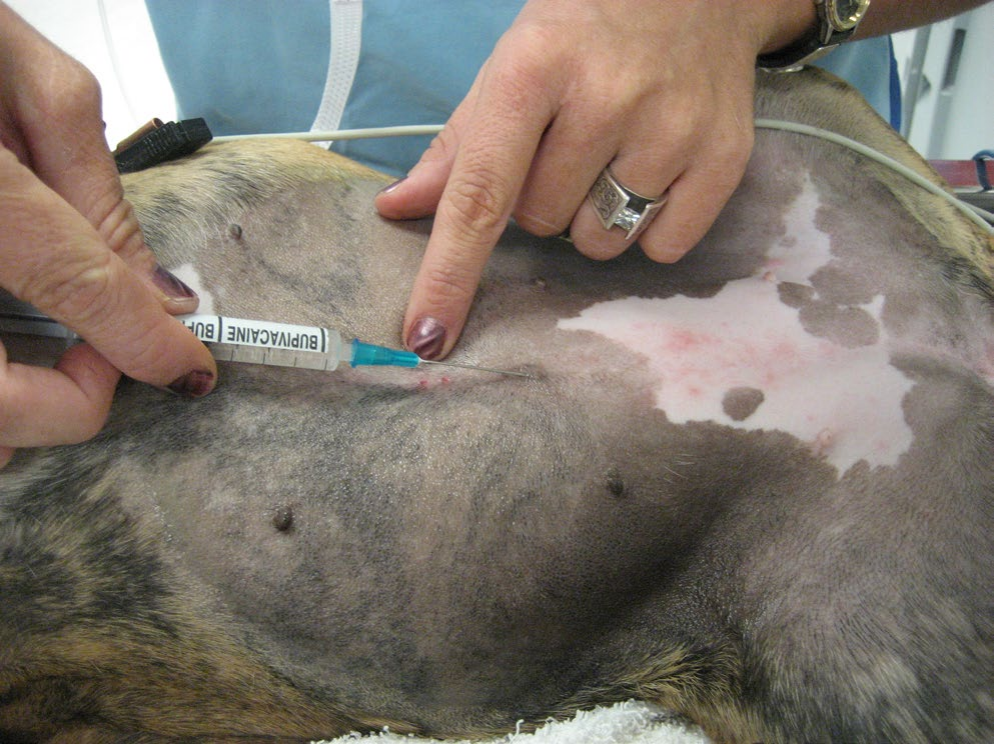
Incisional or field block. This block is being performed after the rough scrub but before the sterile scrub. If performed after the sterile scrub, the person administering the block would wear sterile gloves

BOX 3 **Technique for general tissue infiltration**

▪ Technique: The nerve endings in the tissues are blocked by injecting the drug around or directly into the incision or wound ‘field’. See more on the injection technique for BLIS in Part 1 (Grubb & Lobprise, 2020).
▪ The total injected volume depends on the size of the wound/incision and care should be taken not to exceed the maximum recommended dose for each drug in the species treated. If a larger volume is necessary, drugs, including BLIS, can be expanded with an equal or lower volume of saline.
▪ The body of literature supports that local anaesthetics, including BLIS, injected into an incision/wound do not cause a clinical delay in tissue healing (Abrão et al., 2014; Baxter, Bramlett, One, & Daniels, 2013; Cantatore et al., 2014; Waite, Gilliver, Masterson, Hardman, & Ashcroft, 2010).

Tissues/area desensitized: Dermal and hypodermal layers, and muscle layers depending on depth of injection, in the immediate proximity of the injection site. Use for incision and wound repair.

### 1.4 Oral/dental blocks

CRITICAL POINT for ORAL BLOCKS: Excessive mouth opening should be avoided in cats, as this could compromise the blood flow through the maxillary artery, and no collateral circulation to the cerebrum and retina is present in this species. Severe neurologic deficits and blindness could result from impaired blood flow and ischaemia, particularly when spring‐loaded mouth gags are used to create maximal mouth opening (Barton‐Lamb et al., 2013; Martin‐Flores, Scrivani, Loew, Gleed, & Ludders, 2014; Scrivani, Martin‐Flores, Hatten, & Bezuidenhout, 2013; Stiles, Weil, Packer, & Lantz, 2012). These mouth gags should not be used in cats. In average‐sized adult cats mouth gags that produced a maximal mouth opening of 42 mm between canine teeth, but not mouth opening of 20 or 30 mm, produced blood flow deficits (Martin‐Flores et al., 2014). Thus, mouth gags that do not cause maximal mouth opening are acceptable.

*Infraorbital block* (Gross et al., 2000; Gross, Pope, O’Brien, Dodam, & Polkow‐Haight, 1997) Box 4

BOX 4 **Technique for infraorbital block**

▪ Technique:
  - Push the lip up and palpate the canal opening in the mucosa over the infraorbital foramen in the region dorsal to the third premolar.
  - Insert the needle into the canal and advance the needle no further than a site level with the medial canthus of the eye. Hold the needle and the syringe parallel to the palate and flat against the maxilla. Aspirate, then use a slow, steady injection to deposit the anaesthetic evenly within the canal.
    o In cats (not brachycephalic breeds), the entire infraorbital canal is approximately 4 mm long, ending at the level of the medial canthus. Advancing a needle in the canal is typically not recommended due to the close proximity of the globe.

Tissues/area desensitized: (Kitchell, 1993): When deposited within the canal or approaching the region of the pterygopalatine fossa at the distal aspect of the canal, the premolars, canines and incisors will be desensitized. This block will likely not desensitize the caudal superior alveolar nerve, which branches off the maxillary nerve caudal to the infraorbital nerve and enters the infraorbital canal to innervate the 1^st^ and 2^nd^ molars in dogs and the 3^rd^ and 4^th^ premolars in cats.

*Infraorbital approach to the maxillary nerve block* (Snyder & Snyder, 2013) Box 5

BOX 5 **Technique for infraorbital approach to the maxillary nerve**

▪ Technique: A deep infraorbital block with needle advancement to the level of the maxillary 1st molar has been described (Rochette, 2005) and the use of an intravenous catheter to reach this level has also been reported (Viscasillas, Seymour, & Brodbelt, 2013).
▪ Possible complications include damage to the neurovascular bundle within the canal and intravascular injection. To avoid damage to the globe, this approach is not used in cats and it's use in brachycephalic patients is controversial. Perhaps the approach is unnecessary in these patients due to the short distance necessary for caudal drug diffusion from an injection site at the infraorbital foramen to the entrance of the maxillary nerve into the foramen.

Tissues/area desensitized: Infiltration of the local anaesthetic drug at the caudal aspect of the infraorbital canal may impact the caudal superior alveolar nerve.

*Caudal approach to the maxillary nerve block* (Aguiar, Chebroux, Martinez‐Taboada, & Leece, 2015) Figures 2, 3, 4 and Box 6

**Figure 2:**
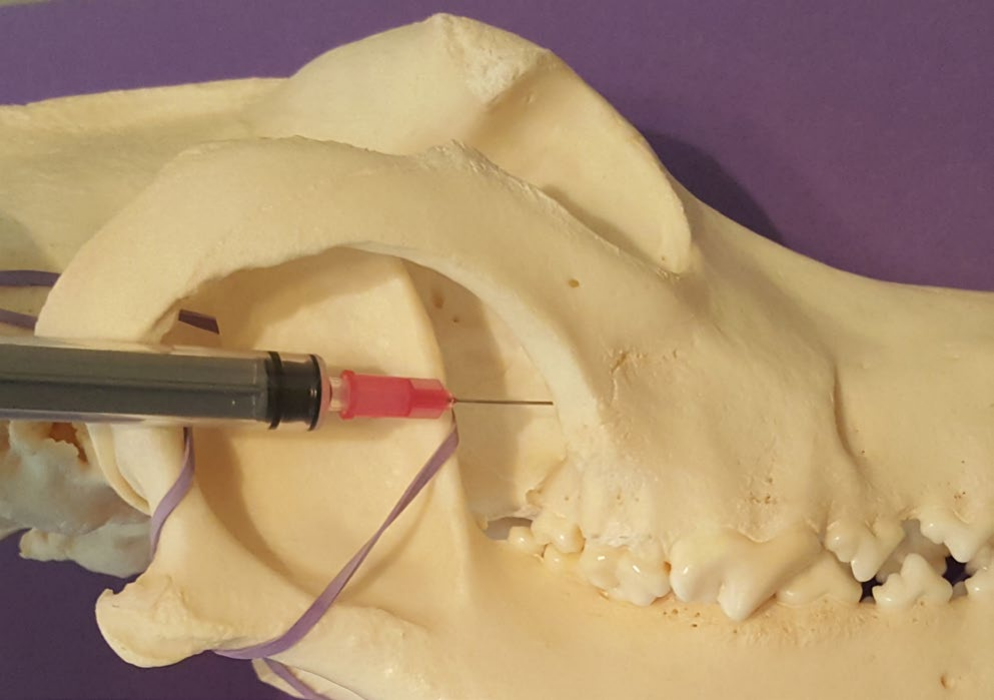
Caudal approach to the maxillary nerve on a dog skull

**Figure 3:**
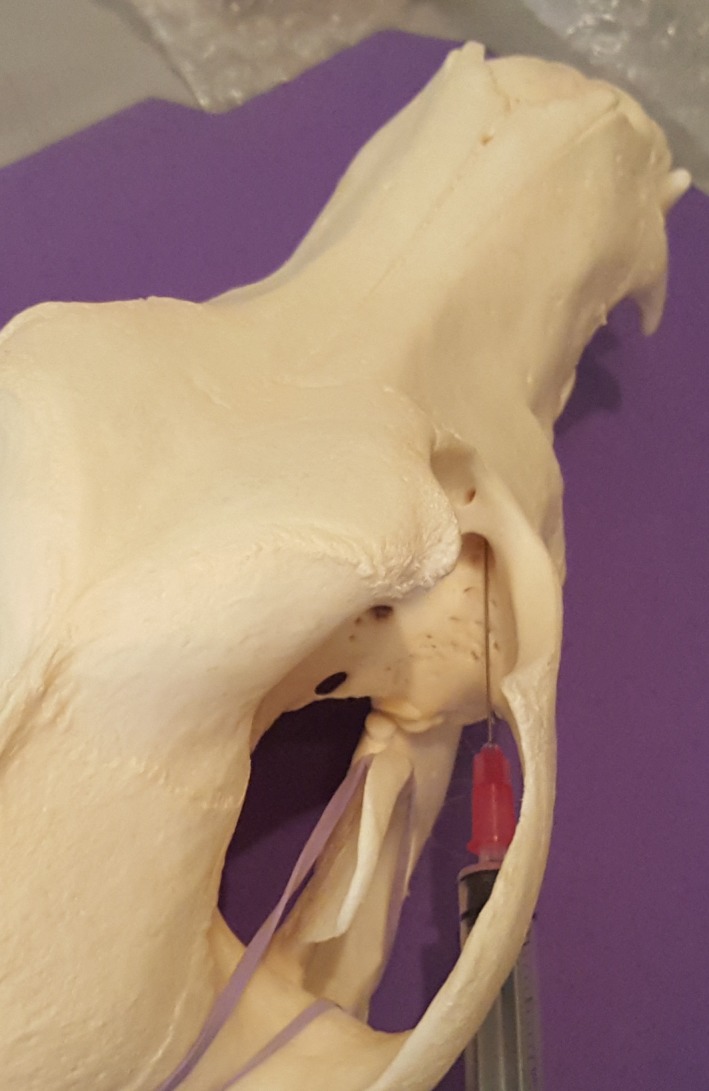
Caudal approach to the maxillary nerve on a dog skull

**Figure 4:**
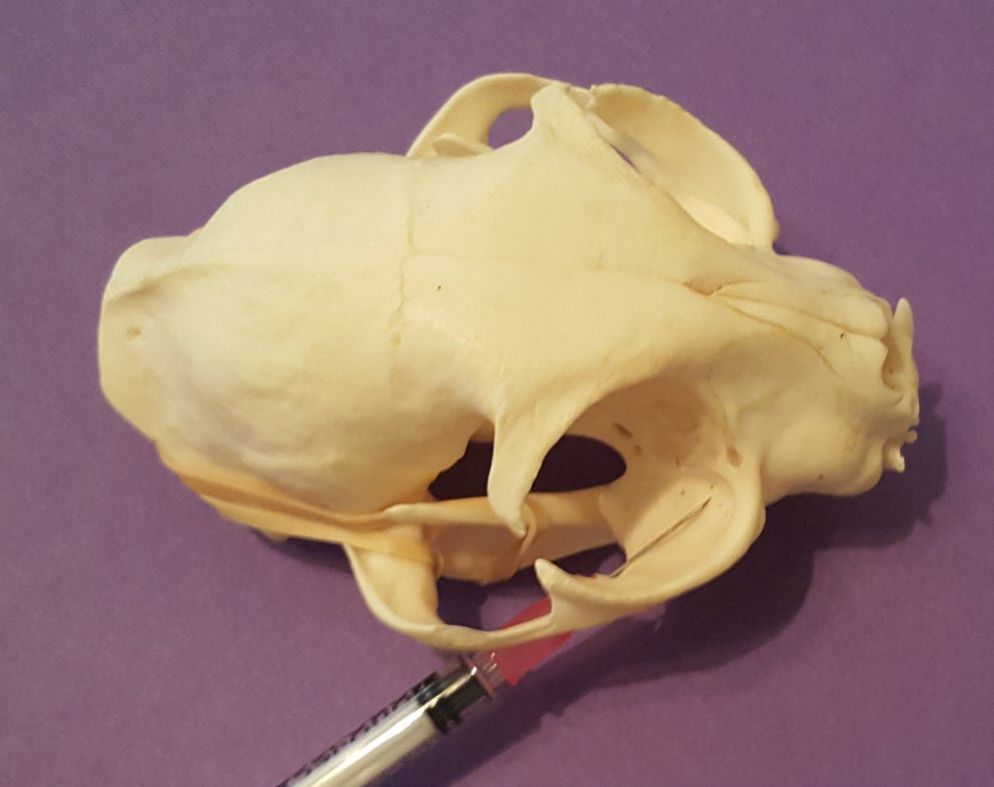
Caudal approach to the maxillary nerve block on a cat skull

BOX 6 **Technique for caudal approach to the maxillary nerve**

▪ Techniques:
  - Percutaneous approach
    o Insert the needle percutaneously just below the ventral border of the midsection of the zygomatic arch, perpendicular to the skin and directed medially (Viscasillas et al., 2013). For medium‐sized dogs, the insertion point is located 0.5 cm caudal to a vertical line drawn from the medial canthus of the eye.
    o Advance the needle into region of the pterygopalatine fossa, aiming slightly rostrally towards the maxillary foramen (Dugdale, 2010). Insert the needle until its full length is in tissue or until it hits bone. Aspirate, inject.
  - Intra‐oral approach
    o Open the mouth and caudally retract the lips.
    o Insert the needle, pointed dorsally, in the mucosa immediately caudal to the mid‐section of the maxillary second molar.
    o Potential complications: Accidental globe perforation has been reported with this approach (Perry, Moore, & Scurrell, 2015), therefore, the needle should not be inserted dorsally more than 2–4 mm, depending on dog/cat size, as recommended by Beckman and Legendre (2002). There is also the potential that the maxillary artery can be perforated.
  - Modified maxillary nerve block – caudal approach. The author prefers this approach as potential injury to the eye is less likely. With enough laxity in the commissure, additional benefits include ability to directly palpate the needle entry point and no need to pass the needle through the skin.
    o Caudally retract the lips to palpate the ventral aspect of the zygomatic arch from the oral cavity or use a modified percutaneous approach.
    o Approach from the caudal direction and keep the needle and syringe in a plane parallel to the palate.
    o Introduce the needle into the mucosa (or skin) ventral and medial to the junction of the zygomatic arch and palatine bones, in a rostral‐medial direction, as if aiming for the opposite nostril. Advance the needle just dorsal to the palatal bone in the direction of the pterygopalatine fossa.
    o Infiltrate the local anaesthetic at the fossa and also while withdrawing the needle in order to block the caudal superior alveolar nerve.

Tissues/area desensitized (Kitchell, 1993): The maxilla and incisive bone with associated soft tissue, upper dental arch, upper lip and nostrils, hard and soft palates.

*Major palatine block* Box 7

BOX 7 **Technique for major palatine block**

▪ Technique:
  - Inject the local anaesthetic halfway between the palatal midline and the dental arcade at the level of the mesial root of the maxillary 1st molar (to the midpoint of the 4th premolar) in the dog and palatal root of the maxillary 4th premolar in the cat (Lantz 2003).

Tissues/area desensitized: Desensitizes the oral side of the hard palate tissues. Used for analgesia for palate surgery.

*Mental nerve block* Box 8

BOX 8 **Technique for mental nerve block**

▪ Technique:
  - Palpate the middle mental foramen ventral to the mesial (rostral) aspect of the 2nd premolar.
    o In cats, the middle mental foramen is at, or immediately caudal to, the apex of the mandibular canine tooth (Lantz 2003).
  - Insert the needle into the soft tissue slightly rostral to that location.
  - In medium to large sized dogs, local anaesthetic drug can be injected directly into the mandibular canal (Lantz 2003) and may provide rostral desensitization sufficient to obviate the need for a complete mandibular block. Guide the needle into the foramen in a cranial to caudal direction with a slight medial angle. Advance as far as possible, approximately 2–4 mm depending upon dog/cat size.
  - In smaller patients, inject local anaesthetic directly under the gingiva immediately rostral to the foramen.

Tissues/area desensitized: An effective mental block ideally reaches the mandibular canal and rostral alveolar nerve branches. Desensitizes the lower lip and rostral intermandibular region (Krug & Losey, 2011) but in a study in dogs, only the 2^nd^, 3^rd^ and 4^th^ premolars were consistently desensitized (Krug & Losey, 2011). In this study, only a portion of the soft tissue was blocked, possibly due to collateral innervation or potentially because the dose used was lower than reported elsewhere (Lemke 2007; Beckman, 2006). Dental tissue desensitization was more consistent (C‐fiber impact) (Ho & DeLuca, 1999).

*Inferior alveolar block* (caudal mandibular; Aguiar et al., 2015) Box 9

BOX 9 **Technique for inferior alveolar block**

▪ Technique: The inferior alveolar nerve is blocked as it enters the mandibular foramen, which is located on the medial (lingual) side of the mandible approximately halfway between the 3rd molar and the mandibular angular process in a dog (Snyder, Snyder, & Beebe, 2019) and halfway to the distal 1st molar in a cat (Lantz 2003).
  - Intraoral approach: With the dog/cat in lateral recumbency and the syringe aimed at the recumbent side, insert the needle through the gingiva near the foramen at the site described above (Rochette, 2005). It is often possible to palpate the foramen, especially in medium to large dogs.
    o According to Goudie‐DeAngelis, Snyder, Raffe, and David (2016) : ‘The mandibular foramen is palpated, whereas the needle bevel is passed along the lingual cortex of the mandibular ramus until it reaches the foramen. The extraoral end of the syringe barrel is then centred over the 1st premolar on the contralateral side of the oral cavity. Aspirate and inject slowly over 30 s’.
  - Extraoral approach; with the dog/cat in lateral recumbency, block the mandible on the nondependent (‘up’) side. Palpate the mandibular foramen intraorally with one hand. With the other hand, insert the needle through the skin close to the medial aspect of the mandible and advance it dorsally in the gingiva to the area of the foramen. Aspirate and inject the anaesthetic (Rochette, 2005).
    o The foramen may be challenging to palpate in smaller dogs and cats (Goudie‐DeAngelis et al., 2016). But palpation of the foramen is not necessary and the approximate area for injection can be determined by using known landmarks. Also described in Goudie‐DeAngelis et al., 2016 are the landmarks: ‘An imaginary line is drawn from the lateral canthus of the eye to the centre of the ventral notch of the mandible. Insert the needle along the lingual cortex, against the periosteum of the ramus with the bevel facing towards the lingual cortical bone. Advance 1/3 of the height of the mandibular body’. The landmarks are the same for either the extra‐ or intra‐oral approach.
    o However, palpation of the foramen may improve block efficacy. Compared to the intraoral approach, this technique offered less accurate placement without digital palpation of the foramen (Goudie‐DeAngelis et al., 2016).
  - Possible complications include:
    Accidental finger needle‐stick. Digital palpation of the foramen may aid needle placement but increases the risk of this complication.
    • Desensitization of the lingual nerve and self‐trauma to the tongue might occur with incorrect placement or use of excessive local anaesthetic drug volume, as evidenced by the one published case report of this complication in which the volume injected was 3x the volume recommended by the authors of this manuscript (Chau, 2017). The authors have not experienced this complication and block both sides simultaneously on a regular basis. If concerned, bupivacaine or ropivacaine could be used to block one side for a longer duration and lidocaine could be used to block the other side. The lidocaine would wear off quickly and decrease the likelihood that the tongue would be desensitized beyond the immediate postoperative phase.
    • Low mandibular nerve (lingual nerve) injury prevalence in humans based on articaine use may be due to a toxicity issue (Hillerup & Jensen, 2006) and has not been reported in veterinary species.

Tissues/area desensitized: The mandible, lower lip and lower dental arch.

### 1.5 Alternative local injection of local anaesthetic drugs

While there are limited studies of alternative techniques in the veterinary literature, application of concepts from human dentistry may provide alternative choices for desensitization of local areas if regional analgesia is not possible. Injection of local anaesthetic in tissue surrounding a maxillary dental surgical field may provide soft tissue desensitization and potentially provide some infiltration into the osseous tissues since the maxillary cortical bone is less dense than that of the mandible (Rochette, 2005). Injection of up to 0.2 ml into the periodontal ligament space of a tooth of a larger dog can be used if extraction is indicated, but recall from Part 1 (Grubb & Lobprise, 2020) of this manuscript that local anaesthetics are less effective in regions of tissue infection (Rochette, 2005).

### 1.6 Other blocks on the cranium

*Retrobulbar block* (Giuliano & Walsh, 2013; Accola et al., 2006; Myrna, Bentley, & Smith, 2010; Shilo‐Benjamini, 2019) Box 10

BOX 10 **Technique for retrobulbar block**

▪ Technique: The technique described here (the inferior‐temporal palpebral retrobulbar block) is used by the author and was deemed the most effective and easiest of 5 different approaches to the retrobulbar block (Accola et al., 2006; Giuliano & Walsh, 2013). Other techniques are described in Shilo‐Benjamini, 2019.
  - Create a slight bend in a 22‐gauge 1.5‐ inch needle.
  - Insert the needle just inside the boney rim of the lower orbit (i.e. on the ocular side of the orbit) half‐way between the lateral canthus and the middle of the lower eyelid. The needle can be inserted either through the skin or with the lower lid pulled ventrally and the needle inserted through the conjunctiva.
  - Slowly advance the needle along the floor of the orbit (sometimes a ‘scraping’ sensation is felt as the needle scrapes the bone) and then dorsally and slightly medially as the needle reaches the back of the globe. Penetration of the orbital fascia can cause a slight ‘pop’.
  - Aspirate and inject slowly. If significant resistance occurs, stop the injection and withdraw the needle slightly and try the injection again. Repeat withdraw/inject until the drug flows easily.
  - BLIS can be used for tissue infiltration postenucleation but has not been described for the retrobulbar technique.
  - Potential injection site complications include penetration of the globe (described as unlikely by Giuliano & Walsh, 2013), intravascular injection (remember to aspirate!), intraneural injection (do not inject if excess pressure), retrobulbar haemorrhage and proptosis, which is more likely with large volumes of anaesthetic used in brachycephalic breeds (Giuliano & Walsh, 2013).

Tissues/area desensitized: Structures of the eye including the conjunctiva, cornea and uvea. Used primarily for enucleation but could be considered for other ocular surgeries. Detailed diagrams are available (Campoy & Read, 2013).

*Auriculotemporal and great auricular nerve block* (Duke‐Novakovski, 2016; Martinez Taboada, 2016). Both references include detailed diagrams. Box 11

BOX 11 **Technique for auriculotemporal and great auricular nerve block**

▪ Technique: Block the *great auricular nerve* by inserting a needle subcutaneously (the nerve is shallow) into the tissue at a point directly ventral to the atlas wing and caudal to the tympanic bulla. The vertical ear canal is palpable slightly rostral to this site and can be used as a landmark.
▪ Technique: Block the *auriculotemporal nerve* by inserting a needle subcutaneously and slightly deeper (if the needle hits the bone, back out to a point ½‐way between the bone and the skin) into the tissue directly above the most caudal aspect of the zygomatic arch, slightly rostral to the vertical ear canal, which is palpable at this site.
▪ Inject approximately 0.5–1.5 ml, depending on dog/cat size, at each location.
▪ Long‐duration blockade is ideal because aural pain can be profound and prolonged.
▪ A potential injection site complication is inability to close the eyelid due to temporary paralysis of the motor portions of the facial and/or temporo‐palpebral nerves (Martinez Taboada 2016). Eye ointment should be used every 2–4 hr until blinking returns.

Tissues/area desensitized: The pinna and the external ear canal. Although few publications exist on the efficacy of this technique, and some publications suggest no advantage of the local block when compared with use of systemic opioids alone (Buback, Boothe, Carroll, & Green, 1996), the author’s clinical experience is that the block is at least partially effective for pain relief and should be included as part of a multimodal analgesic protocol. The author uses the block for ear canal surgeries (total ear canal ablation [TECA], TECA with bulla osteotomy [TECA‐BO], foreign body and growth removals, etc.) and deep cleaning of painful ears. A newly published technique (Stathopoulou, Pinelas, Haar, Cornelis, & Viscasillas, 2018) not yet tried by the author may improve block efficacy. The reviewer of the manuscript agrees with Buback et al., 1996.

### 1.7 Blocks of the thorax and thoracic limbs

**Figure 5:**
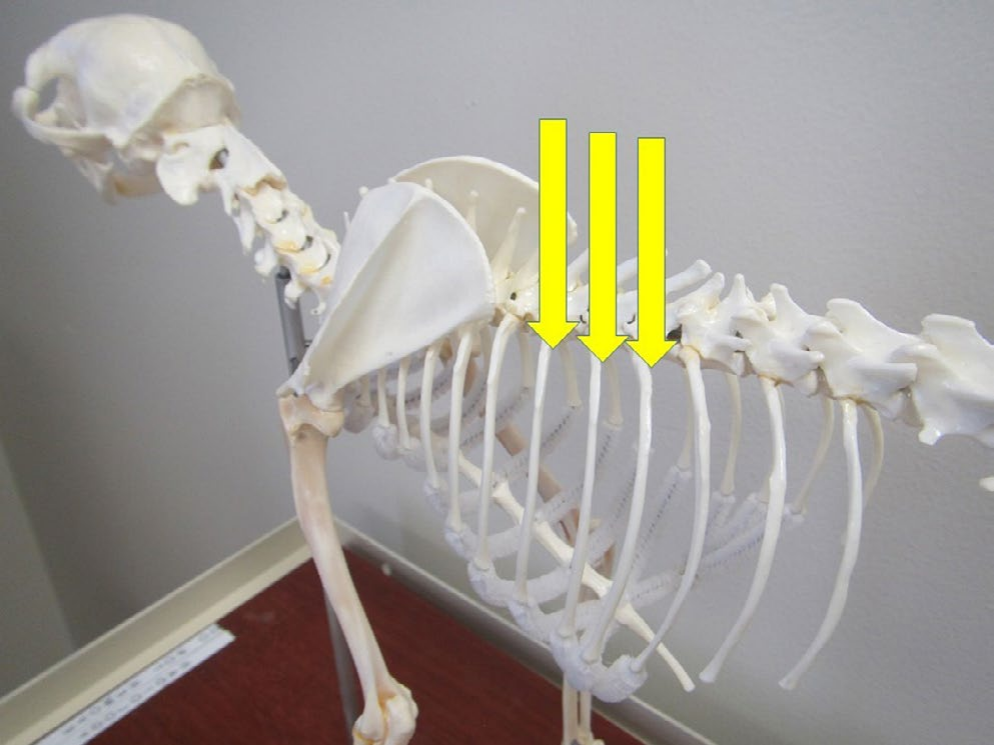
Landmarks for the intercostal block on a dog skeleton. The injection site is indicated by the arrows

*Intercostal block (*Duke‐Novakovski, 2016; Flecknell, Kirk, Liles, Hayes, & Dark, 1991 *)* Figure 5 and Box 12

BOX 12 **Technique for intercostal block**

▪ Technique: Insert the needle into the skin and muscle layers caudal to the proximal portion (i.e. closest to the spinal column) of the ribs in 2–3 rib spaces cranial to and 2–3 rib spaces caudal to the area to be desensitized.
  - Inject between 0.5–2.0 ml per site, depending on dog/cat size.
  - Potential injection site complications include perforation of a vessel and pneumothorax. As with other blocks, intravascular injection is a potential complication and aspiration for blood is required to ensure that the needle is not in the costal artery or vein. Pneumothorax has been reported in human medicine. To decrease the likelihood of causing pneumothorax, the authors recommend using a small gauge needle (22‐G) and ‘walking’ the needle off the caudal edge of the rib, meaning the needle is repositioned using slight changes in the angle of entry (either ‘steeper’ or ‘flatter’) until it slides off the back of the rib. Aspirate for air to ensure that the needle is not in the thorax.
  - If no blood or air is aspirated, inject at this point and continue to inject as the needle is withdrawn out of the muscle layers and skin. If blood or air is aspirated, withdraw the needle slightly and aspirate again. Inject when no blood or air is aspirated.
▪ Liposome‐encapsulated bupivacaine has been used for this block in human medicine (Kelley et al., 2018) resulting in decreased postoperative opioid use for 24‐hr and faster discharge from the hospital compared to patients receiving standard bupivacaine. The authors have used the block in both dogs and cats.

Tissues/area desensitized: Soft tissues of the intercostal space distal to the injection. Used to provide analgesia for thoracic wounds or injury, thoracic drain placement/removal and intrathoracic surgery.

*Blocks to desensitize the forepaws (manus)* Box 13

BOX 13 **Techniques for manus blocks**

▪ Techniques
  - Three‐point block
    o Locate the carpus and accessory carpal pad.
    o Inject 0.1–0.3 ml (depending on dog/cat size) subcutaneously at three sites.
      1 Medial to the accessory carpal pad, blocking the median nerve and palmar branch of the ulnar nerve.
      2 Lateral and proximal to the accessory carpal pad, blocking the dorsal branch of the ulnar nerve.
      3 On the dorsal‐medial portion of the carpus, blocking the superficial branches of the radial nerve.
  - Enomoto four‐point technique for the manus
    o Local anaesthetic is injected in four sites to desensitize the superficial branches of the radial nerve, dorsal branch of the ulnar nerve, median nerve and superficial branch of the palmar branch of the ulnar nerve and the deep branch of the palmar branch of the ulnar nerve.
    o The description of the block is beyond the scope of this paper but the description and corresponding detailed illustrations can be accessed in Enomoto, Lascelles, & Gerard, 2016.
    o Liposome‐encapsulated bupivacaine has been used perineurally for similar blocks in human medicine (Soberon, Ericson‐Neilsen, Sisco‐Wise, Gastanoduv, & Beck, 2016). The Enomoto four‐point block has been used to evaluate analgesia provided by BLIS (NOCITA® product insert website 2018) and is described on the product insert for cats (NOCITA® product insert website 2018).

Tissues/area desensitized: All soft tissue structures of the manus. Used in dogs and cats for painful surgeries or wounds on the paws, such as pad lacerations, torn toenails and removal of foreign bodies.

**Figure 6:**
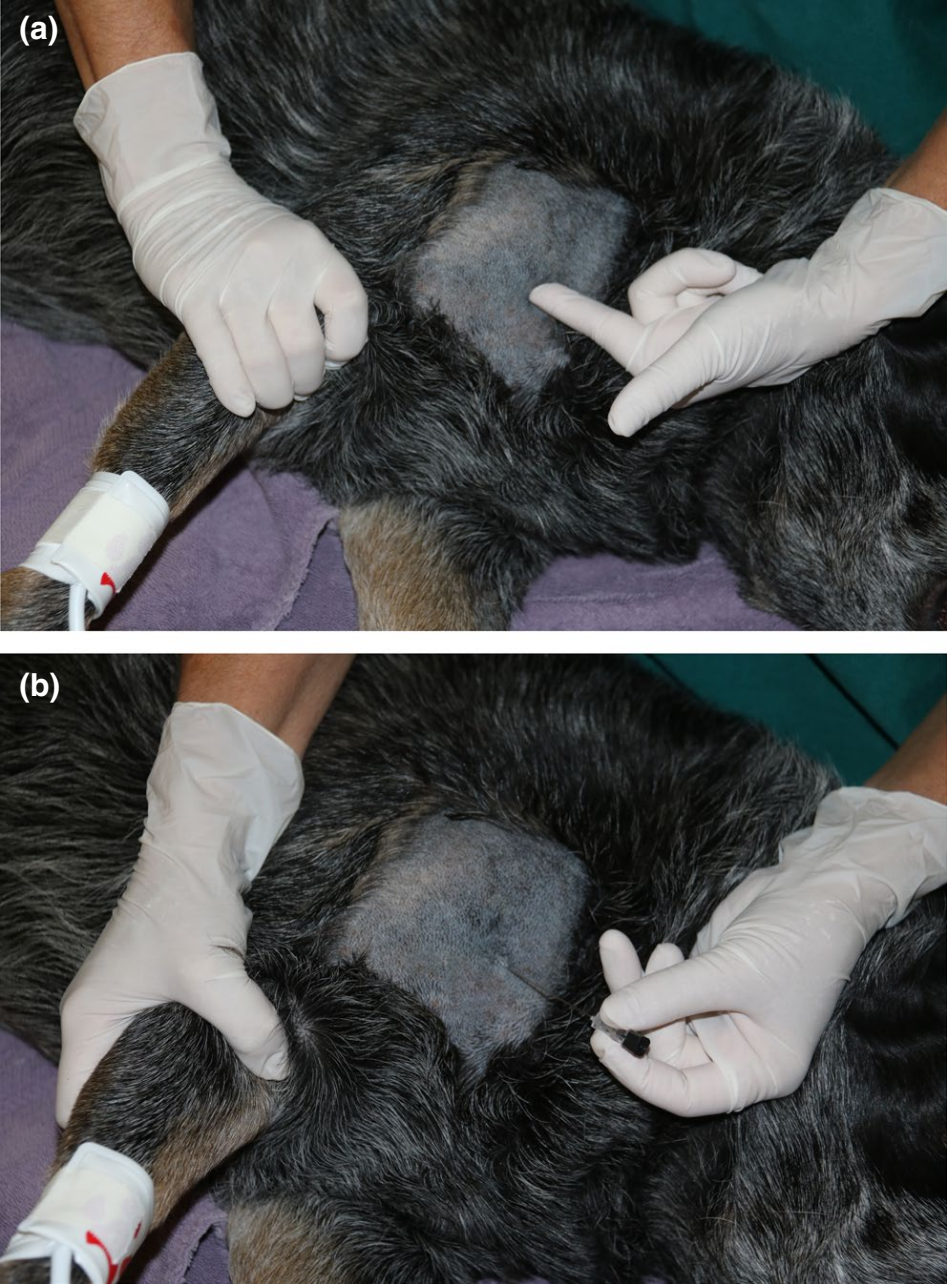
Brachial plexus block landmark at the point of the shoulder (a) and needle insertion (b)

*Brachial plexus blocks *(Duke‐Novakovski, 2016 *; *Mosing, Reich, & Moens, 2010 *; *Wenger, Moens, Jäggin, & Schatzmann, 2005 *; *Duke, Cullen, & Fowler, 1998 *; *Nutt, 1962 *)* Figure 6A&B and Box 14. There are two techniques: (1) Percutaneous 'blind' injection and (2) direct visualization and perinueral injection, which is done only during scapulothoracic dearticulation amputation.

BOX 14 **Techniques for brachial plexus block**

▪ Percutaneous blind injection technique: Locate the acromion, the first rib and the jugular vein.
  - Insert a nerve locator needle or a 2–4‐inch regular needle (a spinal needle will work) on the axial side of the acromion. Aim the needle directly caudally or slightly caudo‐ventrally (parallel to the jugular vein). Keep the needle in a plane sagittal to the rib cage—the tip of the needle may hit the axial side of the scapula. This is normal. Just withdraw slightly and redirect. Stop when the tip of the needle is even with the first rib or the caudal margin of the scapula (these are approximately the same location).
  - Aspirate, then inject 1/3 of the local anaesthetic. Withdraw the needle to the middle of the scapula, and then to a site just before the needle exits the skin, and repeat aspiration and 1/3 injection at both locations.
  - A potential injection site complication is risk of perforating the brachial artery.
  - Use of nerve location or ultrasound guidance may improve block consistency when using this technique (Portelo et al., 2018b).
▪ Direct visualization and perineural injection technique: Inject perineurally as nerves are visualized intra‐operatively during scapulothoracic disarticulation amputation. BLIS would be most effective when injected using this technique. Liposome‐encapsulated bupivacaine is FDA‐approved for perineural injection for a similar block—the interscalene brachial plexus block—in humans.
▪ A technique providing analgesia of the entire forelimb (cervical paravertebral block) has been described (Lemke & Creighton, 2008). Use of a nerve locator or ultrasound improves the accuracy of this block. Other techniques include those published by Skelding, Valverde, Sinclair, Thomason, & Moens, 2018 and Hofmeister, Kent, & Read, 2007.

Tissues/area desensitized: Soft tissues from the distal humerus to the tip of the digits supplied by the musculocutaneous, axillary, radial, median and ulnar nerves. This block does not provide consistent desensitization of the elbow but can be used as part of a multimodal protocol.

*Radius, ulnar, median and musculocutaneous (RUMM) nerve block* (Trumpatori et al., 2010) Box 15

BOX 15 **Technique for radius, ulnar, median and musculocutaneous nerve blockade**

▪ Technique: With the elbow flexed for palpation, locate the sites for injection:
  - Radial nerve on the lateral side of the forelimb: The patient should be in lateral recumbency, lying on the limb that is NOT being blocked. The injection site is on the back of the humerus at the junction of the proximal 2/3 and distal 1/3 length of the humerus (i.e. 2/3 distal to the greater tubercle or 1/3 proximal to the lateral epicondyle). The needle is inserted at the caudal aspect of, and perpendicular to, the humerus. The needle will go through the long head of the triceps (Trumpatori et al., 2010) or between the long and lateral heads of the triceps. Once it hits the humerus, back out slightly and inject 0.1 ml/kg local anaesthetic.
  - Ulnar, median and musculocutaneous nerves on the medial side of the forelimb: The patient should be in lateral recumbency, lying on the limb that IS being blocked. Pull the limb caudally so that it lies behind the upper limb and palpate the brachial artery at mid‐humerus. Insert the needle caudal to the brachial artery and biceps brachialis muscle—both may need to be gently retracted cranially. The needle is inserted at the caudal aspect of, and perpendicular to, the humerus. The needle will go through (Trumaptori et al., 2010) or alongside the long head of the triceps. Once it hits the humerus, back out slightly and inject 0.075 ml/kg local anaesthetic and inject another 0.075 ml/kg as the needle is withdrawn.
▪ There are no specific injection site‐related complications. As with other blocks where injections are made near vessels, venous puncture is uncommon but can occur (Trumpatori et al., 2010).

Tissues/area desensitized: Structures of the distal thoracic limb including the carpus, manus and digits. Detailed diagrams are available (Trumpatori et al., 2010). Used for painful surgeries or injuries distal to the elbow, including down to the manus/pedus. Radius/ulna fracture repair and digit amputation are examples.

### 1.8 Testicular block

*Testicular block* (Perez et al. 2013; Kushnir, Toledano, Cohen, Bdolah‐Abram, & Shilo‐Benjamini, 2017; Huuskonen, Hughes, Estaca, & West, 2013; McMillan, Seymour, & Brearley, 2012) Figure 7A&B and Box 16

**Figure 7:**
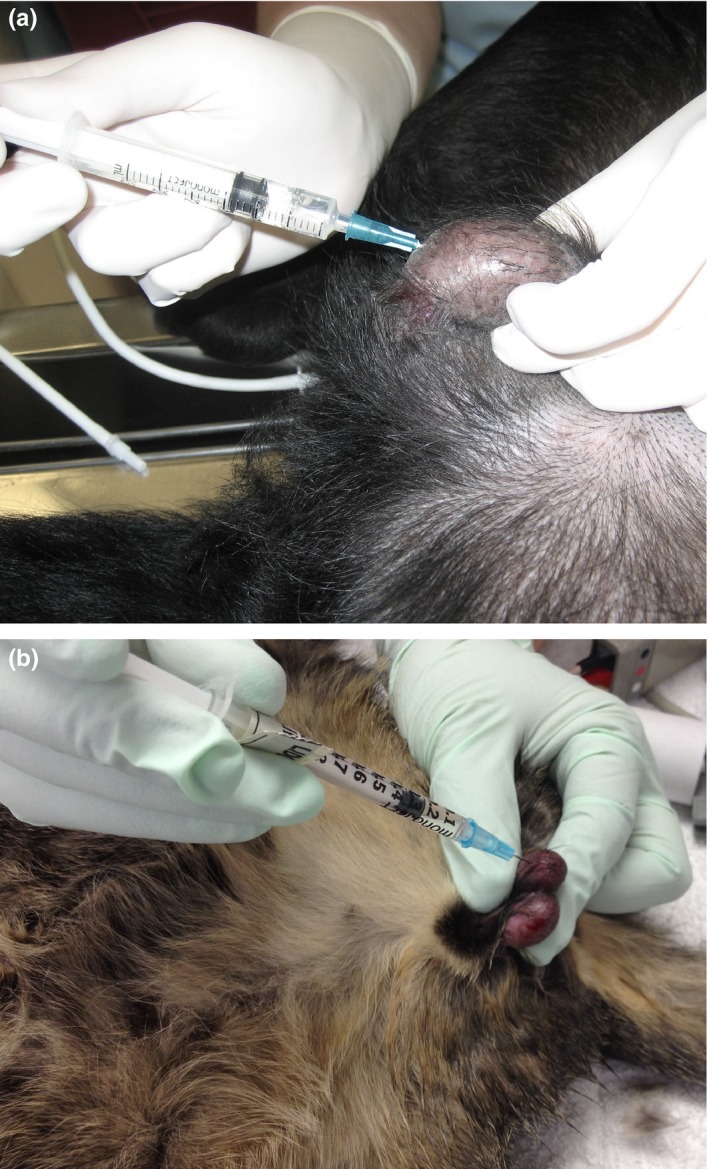
Testicular block in a dog (a) and cat (b)

BOX 16 **Technique for testicular block**

▪ Technique: Insert the needle directly into the testicular body with the needle tip directed towards the spermatic cord.
  - Aspirate, inject half the total recommended dose of bupivacaine, ropivacaine or lidocaine for the species, or the volume that causes increased tissue ‘pressure’ (whichever happens first) into each testicle. Increased tissue pressure is felt as a sudden volume expansion of the testicle.
    ▪ NOTE: Some clinicians use only lidocaine for this block since the testicles are highly vascularized, potentially increasing likelihood of systemic drug uptake. The author uses bupivacaine or ropivacaine, following careful aspiration, because of the longer duration of action. There is no evidence that one drug is better/safer than another so the clinician should use experience and comfort with the drugs to decide which drug to use for this block.
  - The drug migrates up the spermatic cord (Ranheim, Haga, & Ingebrigtsen, 2005) and mitigates intraoperative nociception and postoperative pain from surgical crushing of the cord and associated vessels.
  - In cats, the incision is generally made directly over the testicle and infiltration of the drug should continue as the needle exits the testicle to block the skin and subcutaneous tissue.
  - In dogs, the incision is generally made at a prescrotal site, and local anaesthetic should be injected into skin and subcutaneous tissue at the incision site.
  - Expect to inject between 0.2–2.0 ml per testicle, depending on dog/cat size.
  - Liposomal bupivacaine could be injected directly into the spermatic cord with care to avoid the vasculature but will not migrate to the spermatic cord from the testicle.
  - There are no injection site specific complications but the testicle will often appear ‘bruised’. This is inconsequential since the testicle is being removed.

Tissues/area desensitized: Spermatic cord and associated structures. Provides analgesia for castration.

Intraperitoneal lavage: Tissues/area desensitized: Serosal surfaces in the abdominal cavity including ovarian and uterine tissues associated with OHE. Due to ease of drug administration and number of publications showing efficacy of this block, the author prefers this technique to the mesovarium ligament injection technique.

Mesovarium ligament blockade: Tissues desensitized: mesovarium ligament and associated structures.

### 1.9 Local/regional analgesia for ovariohysterectomy

**Figure 8:**
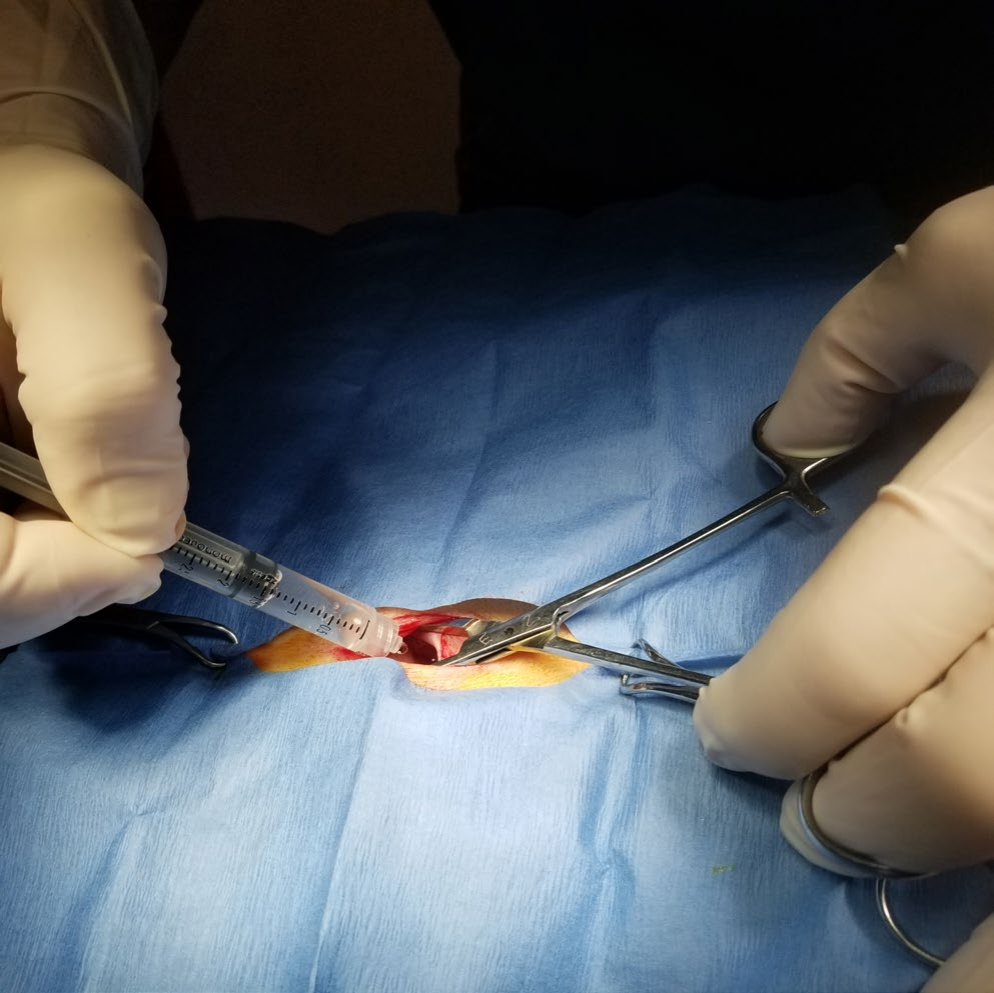
Instillation of local anaesthetics through the ovariohysterectomy incision for lavage of the peritoneal cavity

Two techniques can be used: lavage of the peritoneal cavity with local anaesthetics (Carpenter, Wilson, & Evans, 2004; Wilson, Barnes, & Hauptman, 2004; Benito et al. 2016; Lambertini, Kluge, Lanza‐Perea, Bruhl‐Day, & Kalchofner Guerrero, 2018; Figure 8 and Box 17 and 18) or direct infiltration of the mesovarium ligament (Box 16). For both techniques, the skin and subcutaneous tissues at the incision site should also be blocked.

BOX 17 **Technique for lavage of the peritoneal cavity with local anaesthetics**

▪ Technique: Draw 2–4 mg/kg (cats) and 4–6 mg/kg (dogs) lidocaine OR 1–2 mg/kg (cats) and 2–4 mg/kg (dogs) bupivacaine or ropivacaine into a syringe. If necessary, dilute with sterile saline so that the entire volume is 0.4–0.6 ml/kg.
  ▪ These dosages are conservative. In dogs, 8.8 mg/kg lidocaine OR 4.4 mg/kg bupivacaine (intraperitoneal instillation) plus 2 ml of the same drug as an intra‐incisional injection caused no adverse effects (Carpenter et al. 2004). In this study, the local anaesthetic drug was diluted to a total volume of 0.88 ml/kg. Lidocaine plasma concentrations were below toxic levels when 8 mg/kg was instilled intraperitoneally and 2 mg/kg was injected at the incision site (Wilson et al., 2004).
▪ Instill the mixture in the abdomen immediately after the abdominal wall is incised or immediately prior to closing the abdominal incision to ‘bathe’ or lavage the peritoneal cavity with local anaesthetic drug. Close the incision per routine surgical protocol, leaving the mixture in the abdomen.
▪ Intraperitoneal lavage has been used to control intraoperative nociception and postoperative pain from other abdominal surgeries in human medicine (Boerboom et al. 2018; Ruiz‐Tovar et al., 2016), including caesarean section (Patel et al., 2017). Intraperitoneal lavage is anecdotally used for these surgeries in veterinary medicine, but there are no publications.
▪ BLIS must be injected directly into tissues and, thus, is unlikely be effective for the intraperitoneal technique but can be used to desensitize the incision.

BOX 18 **Technique for direct infiltration of the mesovarium ligament**

▪ Technique: Open the abdomen, elevate either ovary, identify and infiltrate the mesovarium, elevate the opposite ovary, identify and infiltrate the mesovarium, remove the 1st ovary, remove the 2nd ovary, and then proceed with the ovariohysterectomy.
▪ Due to the fact that local anaesthetics are absorbed through mucosal surfaces, ‘dripping’ lidocaine on the ovarian pedicle has been shown to provide analgesia in cats, without the need to expose the mesovarium (Zilberstein, Moens, & Leterrier, 2008). In this study 2% lidocaine 1 mg/kg was injected in the skin, 2 mg/kg was applied topically on each ovary and 1 mg/kg was injected in the abdominal muscular layers. It is likely that use of local anaesthetics in all three sites contributed to the decreased ketamine need in the group that received lidocaine.
▪ The volume injected or ‘dripped’ per side is approximately 0.5 ml in small dogs or cats and up to 3.0 ml per side in large dogs. Volumes up to the maximum recommended dose can be used. Injecting BLIS appropriately into the tissues would be difficult because of lack of visualization at the injection site.

### 1.10 Epidurals

**Figure 9:**
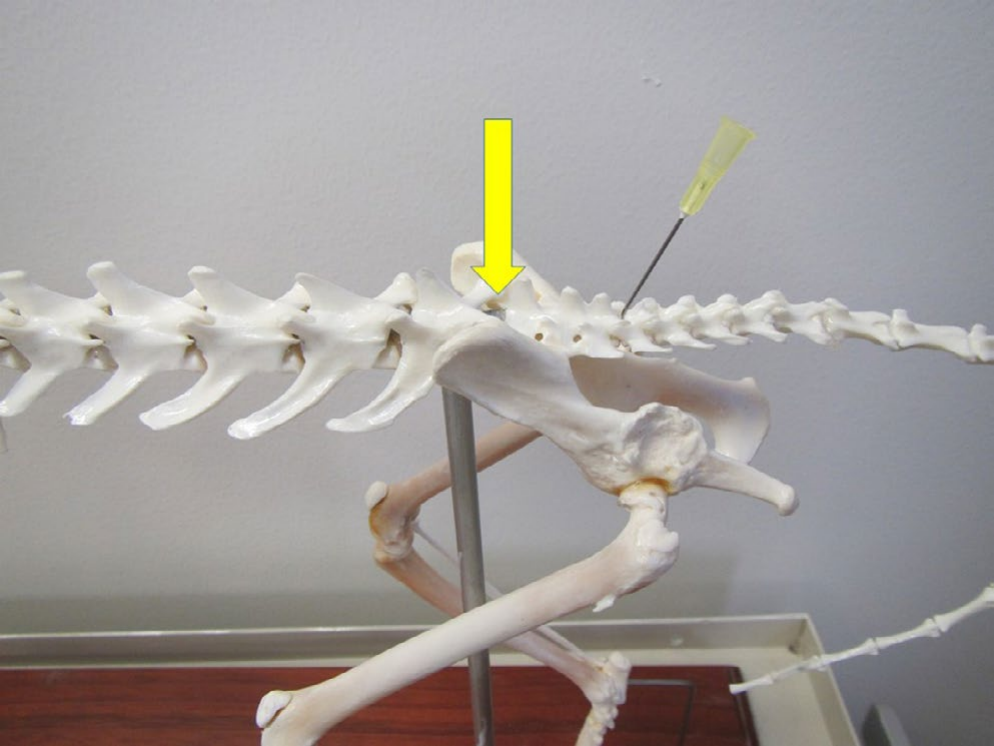
Lumbosacral and sacrococcygeal epidural locations on a dog skeleton: The arrow represents the site for the lumbosacral epidural, which is between the last lumbar vertebra and the cranial edge of the sacrum. The needle represents the site for the sacrococcygeal epidural, which is between the caudal edge of the sacrum and the first coccygeal vertebra

**Figure 10:**
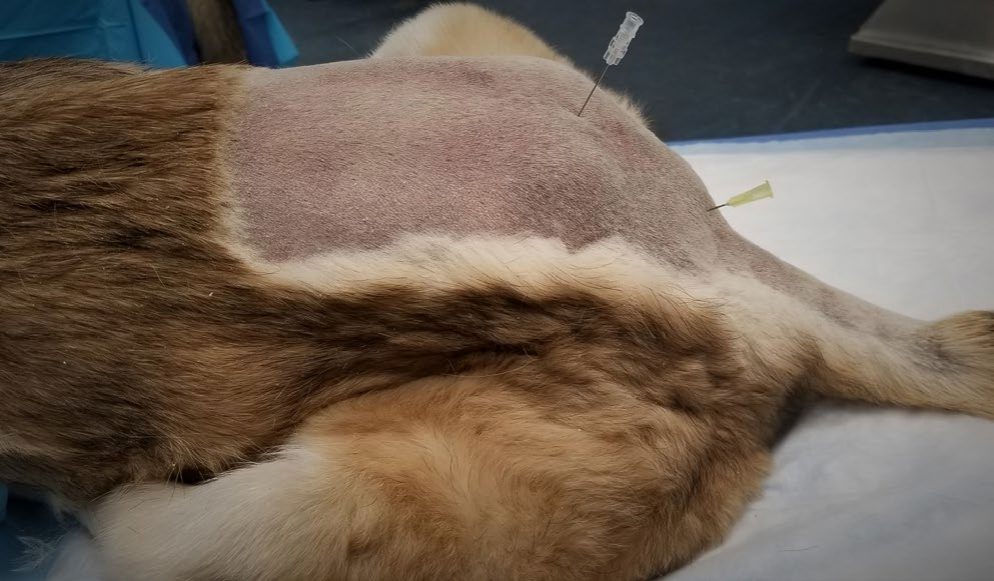
Lumbosacral and sacrococcygeal epidural locations on a cat cadaver: The needle on the left is in the space for the lumbosacral epidural, which is between the last lumbar vertebrae and the cranial edge of the sacrum. The needle on the right is in the space for the sacrococcygeal epidural, which is between the caudal edge of the sacrum and the first coccygeal vertebrae

*Sacrococcygeal or intercoccygeal epidural* Figures 9 and 10 and Box 19

BOX 19 **Technique for sacrococcygeal or intercoccygeal epidural**

▪ Technique: Identify the injection site by moving the tail up‐and‐down (dorso‐ventrally) in a ‘pumping’ motion while palpating the sacrococcygeal region. The first movable space at the caudal end of the sacrum is either the sacrococcygeal or first intercoccygeal space. Either is appropriate and there is no need to determine which site has been identified.
  - Insert a 22‐ or 25‐gauge needle through the skin ON MIDLINE at a 60–90‐degree angle to the skin surface, although a flatter angle (30–45 degrees) may also be used for the initial approach.
  - Proceed slowly until the needle enters the space. Generally the needle will hit the vertebral bone and can be ‘walked off’ the bone, meaning the needle is repositioned using slight changes in the angle of entry (either ‘steeper’ or ‘flatter’) until it enters the space. A ‘pop’ may be felt as the needle penetrates the interarcuate ligament/ligamentum flavum but the lack of a pop should not be interpreted as incorrect needle placement, especially if a regular hypodermic needle is used. These needles are sharper than epidural or spinal needles and may pass through the ligamentum without causing a pop.
  - Inject 0.1 ml/kg, depending on dog/cat size. There should be minimal to no resistance to injection.
  - Do not inject air. Speculatively, an air bubble may cause an incomplete block in such a small space (O’Hearn & Wright, 2011).
▪ Lidocaine, bupivacaine and ropivacaine are appropriate for this block. There is no information for using BLIS and the authors have not used it in this block.
▪ There are no specific injection site related complications. The spinal cord ends at the cranial border of vertebra L7 in large dogs and the lumbosacral junction in small dogs and cats (Wetmore & Glowaski, 2000) so spinal cord damage would not occur. Excessive injectate volume could cause extended cranial migration of the drug, with potential blockade of the motor nerves to the pelvis. However, no pelvic limb dysfunction occurred at a dose of 0.1–0.2 ml/kg local anesthetic administered to cats (O’Hearn & Wright, 2011).
▪ The authors use the block as described in this manuscript, but nerve stimulation can also be used (Otero, & Portela, 2018).

Tissues/area desensitized: Soft tissues of the perineum, tail and sacrum supplied by the pudendal, pelvic and caudal nerves. Provides analgesia for urinary bladder catheter placement (e.g. to relieve urethral obstructions; O’Hearn & Wright, 2011), tail amputations, anal sacculectomies, relief of obstipation, assisted vaginal delivery of puppies or kittens, perineal urethrostomies and other perineal surgeries.

*Lumbosacral epidural* (Garcia‐Pereira, 2018; Valverde, 2008, 2018) Figures 9 and 10 and Box 20

BOX 20 **Technique for lumbosacral epidural**

▪ Technique: Identify the injection site by palpating the lumbo‐sacral junction, felt as a fairly large mid‐line space almost directly in line with the wings of the ilium. The patient can be placed in either sternal or lateral recumbency and the rear legs can either be pulled forward (to increase the distance between the lumbar vertebrae and sacrum, essentially making the space larger) or backward (often making the site easier to palpate in large, overweight dogs).
  - Clip and aseptically scrub this region. Wear gloves. A small drape or sterile glove wrapper used as a drape is recommended.
  - Insert an epidural or spinal needle (e.g. a Touhy needle) into the caudal portion of the LS space with the needle angled at approximately 45° from vertical.
  - Slowly advance the needle until the epidural space is entered.
    o The 'hanging’ drop often works (aspiration of fluid in the hub of the needle as the epidural space is entered).
    o Several ‘pops’ may be felt and can be used to gauge the depth of needle insertion.
    o If bone is encountered, withdraw the needle a few millimetres, redirect slightly (steeper or flatter angle) and reinsert (‘walking’ off the bone). Repeat this process until the needle enters the intervertebral space.
  - STOP advancing as soon as the space is entered and slowly inject the drug.
    o Aspiration at this point is recommended by some, including the author, to ensure that the needle isn't in a vessel (blood would be aspirated) or through the dura (cerebral spinal fluid, CSF, would be aspirated). However, others are concerned that the negative pressure of aspiration could damage delicate dural tissues.
    o If blood is aspirated, the needle can be withdrawn slightly and re‐aspirated until there is no blood present, followed by injection of the drug. Some experts recommend withdrawing the needle completely and re‐starting the injection. The authors use the former option. If CSF is aspirated, the recommendation is to inject only one‐half of the drug volume to avoid excessive cranial migration of the drug with subsequent central nervous system (CNS) depression. Although commonly used, this recommendation is not evidence‐based.
  - A test dose of saline can be injected to ensure that the needle is in the epidural space.
    o The drug should inject easily if the needle is in a space.
    o The most definitive determination that the needle is in the lumbosacral space is to stop injecting and take your thumb off the plunger. The fluid should momentarily continue to flow if the tip of the needle is in the epidural space.
  - If the drug does not inject easily, the likely reason is that the needle was pushed slightly deeper when the syringe was attached. Withdraw the needle 1‐2mm and try again.
  - Once drug has been injected, remove the needle and proceed with surgery.
  - If local anaesthetic drugs have been used, the patient can be positioned with the surgical side down for approximately 5 min so that the drug can ‘bathe’ the nerves of the recumbent limb. If only opioids are used, this manoeuvre will not enhance the analgesia since the site of action is at opioid receptors in the spinal cord, not at local nerves. The efficacy of positioning as just described is questionable.
▪ Opioids are most commonly used but local anaesthetic drugs can be used alone or in conjunction with opioids.
  - 0.1 mg/kg morphine (preservative‐free is gold standard but morphine with preservative can be used). Other opioids provide a shorter duration than morphine but most are acceptable (Valverde, 2008).
  - Dilute to 1 ml/4.5 kg or 0.2 ml/kg total volume with lidocaine, bupivacaine, ropivacaine, sterile saline or sterile water with a total volume of up to 6 mls if local anaesthetics are used (see explanation under ‘complications’).
▪ Morphine is fairly lipid insoluble, and thus stays in the space and provides up to 24 hr of analgesia with minimal systemic uptake. Time to onset of analgesia is 30–60 min. The opioids will cause sensory blockade but are unlikely to cause motor blockade.
▪ Local anaesthetics have an onset and duration of action similar to their onset and duration at any other site. Local anaesthetics enhance the analgesic effects of the blockade (Kona‐Boun, Cuvelliez, & Troncy, 2006). They can cause motor blockade, however, the motor effects are generally minimal or absent by the time the patient has recovered from anaesthesia to the point that it is ambulatory.
▪ Specific complications include ineffective or incomplete block (the most common ‘complication’), epidural haematoma or abscess, or hyperalgesia (very rare). An excessive volume of local anaesthetic could migrate cranially to the point of blockade of the nerves controlling the diaphragm, with subsequent impairment of ventilation. The recommendation not to exceed 6‐mls total volume of local anaesthetics is made to prevent this complication. Urinary retention may occur but is of low incidence and bladders are easily expressed (Kalchofner Guerrero et al., 2014; Troncy et al., 2002). Delayed hair regrowth also occurs at the shave site (Kalchofner Guerrero et al., 2014; Troncy et al., 2002). This has nothing to do with epidural injection of drugs but is due to fact that shaved hair grows back more slowly at the lumbosacral region than any other region (Diaz, Torres, Nogueira, Gilbert, & Jessen, 2006). Contraindications include bleeding disorders (to prevent haematomas) and skin disease over the LS space (to prevent abscesses). Abnormal pelvic anatomy (either from congenital lesions or trauma) may make epidurals difficult.
▪ Epidural catheters can be placed in larger dogs and maintained for several days to allow continuous or intermittent delivery of analgesic drugs (Swalander, Crowe, Hittenmiller, & Jahn, 2000).

Tissues/areas desensitized: All structures caudal to the injection site, including structures of the pelvic limb, perineum and tail. The cranial extent of desensitization is generally T13 if using an injectate volume of 0.2 ml/kg. Larger volumes, or use of an epidural catheter, can extend the desensitized area further cranially. Used for analgesia for abdominal surgeries, pelvic limb surgeries including orthopaedics and can be used for the same perineal/urogenital procedures as those listed for the sacrococcygeal epidural.

### 1.11 Other Blocks of the pelvic limbs

*Blocks to desensitize the rear paws (pedus)* Box 21

BOX 21 **Techniques for blocking the pedus**

▪ Ring block: Inject local anesthetic drugs subcutaneously across the entire dorsum and entire ventrum of the pedus at a location approximately in the middle (from proximal to distal) section of the manus. The two subcutaneous injections will make a 'ring' of local anesthetic blockade.
▪ Enomoto technique for the pedus
  - Two blocks are proposed from deposition of dye near specific nerves. The distal crus block includes the tibial nerve, common fibular (peroneal) nerve and saphenous nerve. The distal pes block includes two branches of the common fibular (peroneal) nerve (the superficial fibular [peroneal] nerve and the deep fibular [peroneal] nerve), tibial nerve and saphenous nerve.
  - The description of the block is beyond the scope of this paper but the description and corresponding detailed illustrations can be accessed in Enomoto et al., 2017.

Tissues/areas desensitized: All soft tissue structures of the pedus. Used in dogs and cats for painful surgeries or wounds on the paws, such as pad lacerations, torn toenails and removal of foreign bodies. 

**Figure 11:**
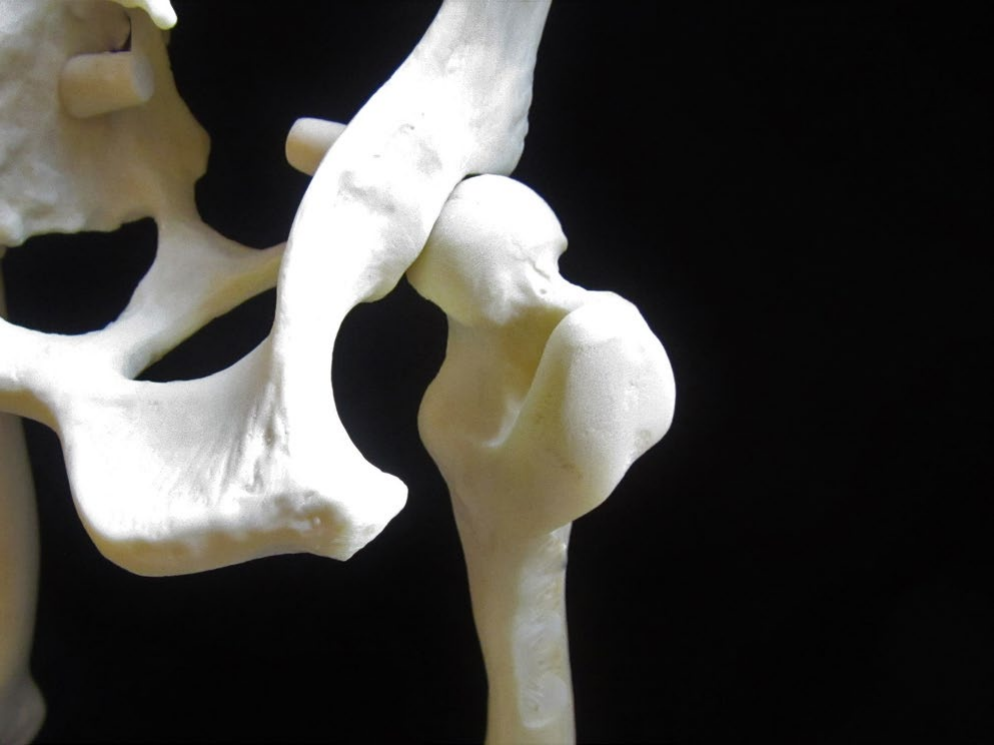
Landmarks for the sciatic nerve block on a model of a dog pelvis/femur. On the left is the ischial tuberosity and on the right is the greater trochanter of the femur. The injection is made 1/3 of the distance from the greater trochanter on a line that would connect the greater trochaner and the ischial tuberosity. Used for orthopedic and soft tissue surgeries of the pelvic limb.

*Sciatic/femoral block (*McCally, Bukoski, Branson, Fox, & Cook, 2015 *; *Caniglia et al., 2012 *; *Campoy, Martin‐Flores, Ludders, Erb, & Gleed, 2012 *;* Gurney & Leece, 2014 *)* Box 22 and Figure 11.

BOX 22 **Technique for blocking the sciatic and femoral nerves**

▪ Although no comparative data are published, a nerve finder or ultrasound guidance is likely to improve block accuracy, especially of the femoral nerve (Campoy & Read, 2013; Otero & Portelo, 2018; Portela et al., 2018a).
▪ The technique described here is used by the author. Reviews of this and other techniques are published in Gurney & Leece 2014, Campoy & Read, 2013 and Otero & Portela, 2018.
  - Sciatic nerve Figure 11
    o The nerve is easy to locate because it lies in the groove created by the greater trochanter of the femur and the ischial tuberosity.
    o Inject approximately 0.1 ml/kg local anaesthetic 1/3 the distance from the greater trochanter on a line with the ischial tuberosity.
  - Femoral nerve (Detailed diagram available [Campoy & Read 2013])
    o The nerve lies in the ‘femoral triangle’ on the inside of the thigh, bordered dorsally by the rectus femoris muscle, cranially by the caudal portion of the sartorial muscle, and deep by the iliopsoas muscle.
    o Locate the femoral artery and keep a finger on the artery to avoid accidental penetration. Direct the needle dorso‐medially to the artery to a depth equal to that of the artery.
    o A potential complication is perforation of the femoral artery so, as with all blocks, aspirate. Inject approximately 0.2 ml/kg local anaesthetic.
    o The safety study for FDA approval of BLIS in cats utilized a suprainguinal approach to the femoral nerve at an increased dosage (NOCITA® product insert website). Liposome‐encapsulated bupivacaine has been used for femoral nerve blocks in humans (Snyder, Scheuerman, Gregg, Ruhnke, & Ellen, 2016).

Tissues/area desensitized: Used together, these blocks will desensitize structures of the rear limb from the distal femur through the digits.

## 2 CONCLUSION

Local and regional blocks are safe and efficacious in dogs and cats when performed correctly using appropriate drug dosages, as described in this review. Local anaesthetic drugs can be injected directly into the tissues to provide analgesia for surgical incisions or wounds, or can be injected perineurally to provide analgesia for a wide variety of painful conditions. Due to the potential to provide profound analgesia and the high safety margin (when used correctly) of this class of drugs, local anaesthetics are recommended as part of the analgesic protocol in the majority of patients undergoing surgical procedures or suffering traumatic injuries. There are numerous local and regional blocks described for use in dogs and cats, thus providing the practitioner with numerous opportunities to block nociception and pain transmission and provide more profound pain relief than is provided with the use of systemically administered drugs alone.

## CONFLICT OF INTEREST

Although manuscript preparation was supported by Aratana Therapeutics, who manufactures liposome‐encapsulated bupivacaine (NOCITA®), the authors feel that the information in the manuscript is a balanced view of the use of all local anaesthetics with detailed information on liposome‐encapsulated bupivacaine since it is a new product. The authors have no other conflicts.

## ETHICAL STATEMENT

The authors confirm that the ethical policies of the journal, as noted on the journal's author guidelines page, have been adhered to. No ethical approval was required as this is a review article with no original research data.
